# Informing healthcare operations with integrated pathology, clinical, and epidemiology data: Lessons from a single institution in Kenya during COVID-19 waves

**DOI:** 10.3389/fmed.2022.969640

**Published:** 2022-09-06

**Authors:** Allan Njau, Jemimah Kimeu, Jaimini Gohil, David Nganga

**Affiliations:** ^1^Department of Pathology and Laboratory Medicine, Aga Khan University Hospital, Nairobi, Kenya; ^2^Department of Nursing, Aga Khan University Hospital, Nairobi, Kenya; ^3^Department of Pharmacy and Therapeutics, Aga Khan University Hospital, Nairobi, Kenya

**Keywords:** integrated data, pathology, pharmacy, laboratory, nursing, epidemiology, COVID-19, Kenya

## Abstract

Pathology, clinical care teams, and public health experts often operate in silos. We hypothesized that large data sets from laboratories when integrated with other healthcare data can provide evidence that can be used to optimize planning for healthcare needs, often driven by health-seeking or delivery behavior. From the hospital information system, we extracted raw data from tests performed from 2019 to 2021, prescription drug usage, and admission patterns from pharmacy and nursing departments during the COVID-19 pandemic in Kenya (March 2020 to December 2021). Proportions and rates were calculated. Regression models were created, and a *t*-test for differences between means was applied for monthly or yearly clustered data compared to pre-COVID-19 data. Tests for malaria parasite, *Mycobacterium tuberculosis*, rifampicin resistance, blood group, blood count, and histology showed a statistically significant decrease in 2020, followed by a partial recovery in 2021. This pattern was attributed to restrictions implemented to control the spread of COVID-19. On the contrary, D-dimer, fibrinogen, CRP, and HbA1c showed a statistically significant increase (*p*-value <0.001). This pattern was attributed to increased utilization related to the clinical management of COVID-19. Prescription drug utilization revealed a non-linear relationship to the COVID-19 positivity rate. The results from this study reveal the expected scenario in the event of similar outbreaks. They also reveal the need for increased efforts at diabetes and cancer screening, follow-up of HIV, and tuberculosis patients. To realize a broader healthcare impact, pathology departments in Africa should invest in integrated data analytics, for non-communicable diseases as well.

## Introduction

Healthcare demands and the infrastructure required to meet those demands have become increasingly complex in modern times. In fact, these have radically changed during the coronavirus disease 2019 (COVID-19) pandemic, especially during the peaks or surges. To date, over 535 million cases and over 6.3 million deaths have been recorded (The Johns Hopkins Coronavirus Resource Center^1^ [Accessed 13 June 2022]). In Africa, although it initially appeared that the disease had an attenuated course in terms of cases and mortality, the impact is by no means insignificant (1–4). Since the definition of the first COVID-19 case in Kenya, March 2020, five waves have occurred, resulting in over 5,600 deaths (Ministry of Health, Republic of Kenya^2^ [Accessed 13 June 2022]). Similar to global observations, the implications of these surges have been a massive influx of COVID-19 patients, some of whom required intensive care, marked pressure on pathology services, delivery of nursing care, supply of therapeutics, excess mortality, and a plethora of downstream healthcare needs generated by the pandemic (5–8).

From a pathology perspective, the sheer number of severe acute respiratory syndrome coronavirus 2 (SARS-CoV-2) tests, in the form of reverse transcription polymerase chain reaction (RT-PCR), in combination with all the logistical complexities of high volume sample and reagent management, introduced enormous pressure in laboratories. Important to note that, in low- and middle-income countries (LMICs), particularly in Africa, only a few laboratories had existing molecular diagnostic capacity, and hence, these had to be developed in record time to meet the clinical demand for testing (9, 10). There was also a ripple effect in all disciplines of pathology; clinical chemistry, microbiology, hematology, and anatomic pathology. A South African study looking at the short-term effects of test ordering coming from routine follow-up of patients with communicable and non-communicable diseases (NCDs) noted a significant drop (up to 80% for some tests), in orders for creatinine, lipids, HbA1c, thyroid-stimulating hormone (TSH), and free triiodothyronine (fT3), as a consequence of lockdown (11). Another study observed increased ordering of procalcitonin and lactate dehydrogenase, driven by a clinical demand for prognostication purposes. On the contrary, volumes in hematology and virology declined (12). In South Australia, a decline in almost all pathology tests except for molecular microbiology was noted. In particular, troponin, a marker of acute coronary syndrome (ACS) care, declined, begging the question of whether this was caused by a reduced incidence of ACS or because of hospital avoidance (13).

Prescription drug utilization also changed during the pandemic, and a particular note has been made of antibiotics: β-lactams, macrolides, fluoroquinolones, glucocorticoids; dexamethasone, prednisolone, and methylprednisolone were used especially for moderate and severe COVID-19 (14). Newly designed or repurposed therapeutics to treat severely ill COVID-19 patients were quickly adopted or as soon as clinical trials reporting variable degrees of efficacy were reported: monoclonal antibodies; SARS-CoV-2 neutralizing types, and anti-inflammatory types such as tocilizumab, an anti-interleukin-6 (IL-6) receptor antibody, and remdesivir, an antiviral agent (15, 16). In Kenya, remdesivir became available around June 2020. Tocilizumab had been in use for the treatment of severe rheumatoid arthritis; its use, however, increased significantly with the pandemic, perhaps also because our institution was a study site for the EMPACTA trial which evaluated the efficacy of tocilizumab in hospitalized patients with COVID-19 pneumonia (17).

Closely related to therapeutics was the unmet need for in-patient care. This includes not just intensive care unit (ICU) beds but also increased demand for intensivists, nursing care, and personal protective equipment (7, 18, 19). And, all this in a background of other communicable and NCDs, medical and surgical healthcare needs, that now had to be conducted with heightened infection control protocols. In this volatile public health climate, data and indicators related to the pandemic such as positivity rate, incidence rates, clinical trials hospitalization rates, case-fatality ratio, and vaccination coverage published in publicly available national or global platforms proved to be extremely useful for both patient treatment and policy or guideline making.

Having experienced numerous healthcare delivery challenges caused by the pandemic, we sought to identify evidence-based tools that may be useful for healthcare planning at an institutional level. We become aware that, although a large amount of data has been captured in the institutional healthcare information systems, analysis of these data for evidence-based planning was lagging. Pathology and epidemiology data appeared to be central; however, an integrated approach encompassing data from clinical care teams, pharmacies, and therapeutics promised greater insights. In addition, guidelines or policies based on global data required contextualizing using local data for meaningful local interventions, not to mention that institutional and local data are tributaries of global data. Given that any policy or guideline has to be changed constantly with the evolution of the pandemic and that they have significant public health and resource ramifications, reliance on robust and accurate data is crucial. The aim of this study, therefore, was to demonstrate how departments of pathology and clinical care teams can play a broader healthcare role by analyzing and describing the patterns of utilization of healthcare variables. Although the focus of the study was on COVID-19, the approach is applicable to other health problems for which we need to understand the interaction of science, policy, socioeconomic factors, and health-seeking and delivery behavior in Africa.

## Materials and methods

### Study setting and design

This was a cross-sectional, multidisciplinary study including the departments of Pathology, Nursing, and Pharmacy, at the Aga Khan University Hospital, a 280 bed tertiary, teaching, and referral hospital in Nairobi, Kenya. A temporary field hospital was set up to increase the capacity for taking care of COVID-19 patients, and this brought the bed capacity for COVID-19 patients to 88, of which 11 were ICU beds. Our objective was to explore data analytics for describing the patterns of healthcare utilization using indicators from the laboratory, pharmacy, and clinical care teams in an integrated fashion. The rationale for the study was that such utilization patterns would not only feed into larger national and international data sets that are used for modeling and projections but would also be critical in planning for near and intermediate future healthcare needs. Although the focus was on data around COVID-19, the bigger picture we envisioned was that African countries would use a similar approach to develop additional tools for a more efficient allocation of scarce health resources and improved healthcare operations. Given that many health problems, from seasonal infections to cancer and to metabolic diseases, that come to the attention of healthcare workers be they physicians or nurses will require a laboratory test and/or prescription, a steady source of data is guaranteed.

### Data retrieval

We began by developing a list of both laboratory tests and prescription drugs whose turnover was perceived or projected to change as the pandemic evolved. These included those that were flagged for stock outs, on the one extreme, or as slow-moving on the other. From the existing hospital information system, queries to extract raw data were generated. The time frame was March 2019 to December 2021, with 2019 pre-pandemic data serving as a baseline, and 2020 to 2021 as 2 years of ongoing COVID-19 pandemic data. Laboratory data retrieved were monthly clustered tallies for tests including malaria parasite, D-dimer, fibrinogen, C-reactive protein (CRP), procalcitonin, serum sodium, HbA1C, HIV viral load, blood group, blood count, cervical smear (PAP smear), histology, blood culture, Mycobacterium tuberculosis (MTB), rifampicin (RIF) resistance (MTB/RIF), SARS-CoV-2, RT-PCR, and summation of all laboratory tests. The list of tests was representative of all sections of the laboratory: clinical chemistry, hematology, blood bank, microbiology, molecular, and histology. Similarly, from the pharmacy, monthly clustered tallies of issued prescriptions for azithromycin, dexamethasone, tocilizumab, enoxaparin, fentanyl, remdesivir, and piperacillin/tazobactam (PIPZO) were retrieved. These drugs were selected based on the perceived changes that would occur in their utilization. As highlighted in the introduction, dexamethasone, remdesivir, and other anti-inflammatory agents were variably adopted for the management of severe COVID-19. In addition, we were also keen on knowing what the trends of antibiotic utilization would be, and therefore, we included azithromycin and PIPZO in our analysis. We did not delve into the area of rational antibiotic use in this paper. Pharmacy data were retrievable from 2020, and, hence for baseline, data for 3 months prior to the full-blown pandemic in the country (January to March 2020) were used. From the nursing department, we obtained bed occupancy for COVID-19 patients including the intensive care unit (ICU).

### Data analysis

Laboratory data were in the form of raw numbers of each test performed and were clustered in monthly tallies. The institutional COVID-19 positivity rate was continuously monitored as a 7-day rolling positivity rate. In this manuscript, this was collapsed into a monthly rolling positivity rate. Pharmacy data were also collected as monthly tallies; however, due to the multiple dosage formulations available for each drug, standardized units were created. As an example, dexamethasone may exist in 2, 4, or 6 mg formulations, and to standardize, all issued doses were converted to 6 mg units. The standardized units for the other prescription drugs were as follows: enoxaparin, 80 mg; azithromycin, 500 mg; remdesivir, 100 mg; tocilizumab, 80 mg; PIPZ0, 4.5 g; fentanyl, 100 mcg. From the clinical care teams, daily censuses for in-patient and ICU COVID-19 patients were collected and monthly averages were calculated. Data analysis and visualization were performed using Microsoft Excel version 16 (Microsoft Corporation) and RStudio, running R software for data analysis, version 4.1.2 (Boston, MA). The monthly mean number of tests for each of the selected tests in 2020 and 2021 was compared to the corresponding baseline mean (2019 data). To determine the statistical difference in the means, a *t*-test (unpaired and unequal variance) was calculated, and *p*-values < 0.05 were considered to be statistically significant. With regard to prescription drugs, the relationship between the number of units for each drug was plotted against the COVID-19 positivity rate, and non-linear regression models were created. Best fit models were determined using visual inspection and partial F-test compared to the linear model, whereby *p*-values > 0.05 were considered to significantly improve model fit. Finally, the COVID-19 positivity rate was plotted superimposed over percentage ICU hospitalization trends, selected laboratory tests, and prescription drug utilization.

### Ethics consideration

This study was approved by the Institutional Ethics Review Committee of Aga Khan University Hospital, Nairobi [2021/IERC-87(v2)]. The study only looked at institutional data sets from the health and laboratory information system. Patient information, charts, records, or samples were not the subject of this study, nor were they investigated.

## Results

**Figure 1:**
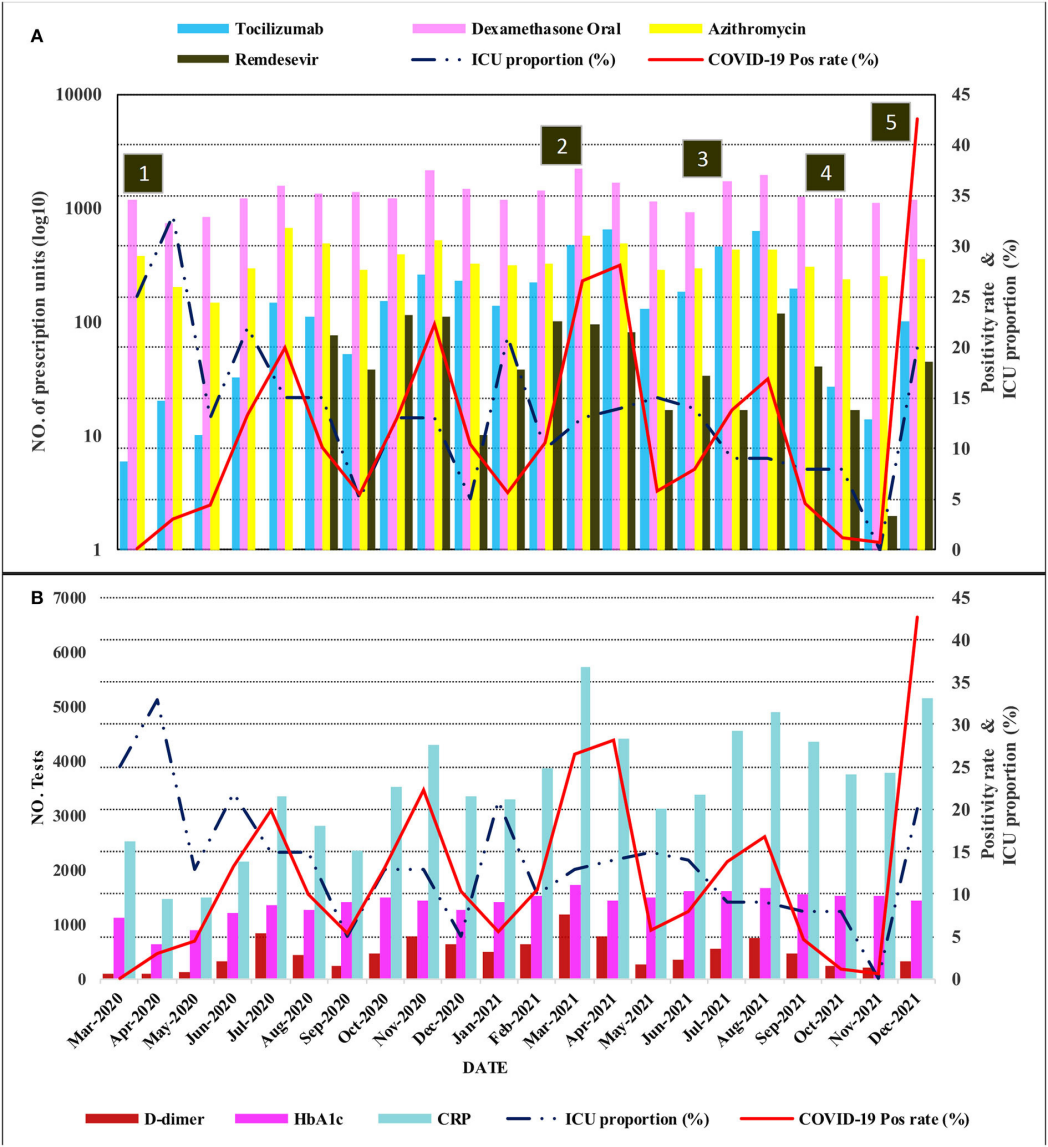
Compound plot showing the trends in the positivity rate and proportion of patients in ICU as line graphs on the right vertical axes. The movement prescription drugs and laboratory tests are plotted on the left vertical axes using bar charts in **(A,B)**, respectively. The prescription drug scale is log10 transformed. The horizontal axis (time series from March 2020 to December 2021) is common for the vertically aligned plots (ICU, intensive care unit; CRP, C-reactive protein). **Annotation in numbered squares**: (1) Impositions of restrictions and curfew, (2) start of vaccination and rise of predominantly *Alpha* variant, (3) *Delta* variant wave, (4) Lifting of restrictions and curfew, (5) *Omicron* variant wave.

By December 2021, close to 118,000 SARS-CoV-2 PCR tests had been performed. Five distinct waves with the zenith points in the months of July 2020, November 2020, March 2021, August 2021, and December 2021 occurred as shown in Figure 1. The characteristics of this trend matched very well with the national tracking which contained a larger sample size, giving external validity to the data set. Genomic epidemiology studies in the country revealed that the first two waves were predominantly caused by the original SARS-CoV-2 strain, while the third, fourth, and fifth were caused by the *Alpha, Delta*, and *Omicron* variants, respectively (20). The trend of the percentage of patients in ICU generally followed the positivity rate with less accentuated peaks. The initial ICU proportion in March and April 2020 was high (33%). This was thought to be due to the relatively small number of COVID-19 patients at the beginning of the pandemic, as opposed to overrunning of the bed capacity.

**Figure 2:**
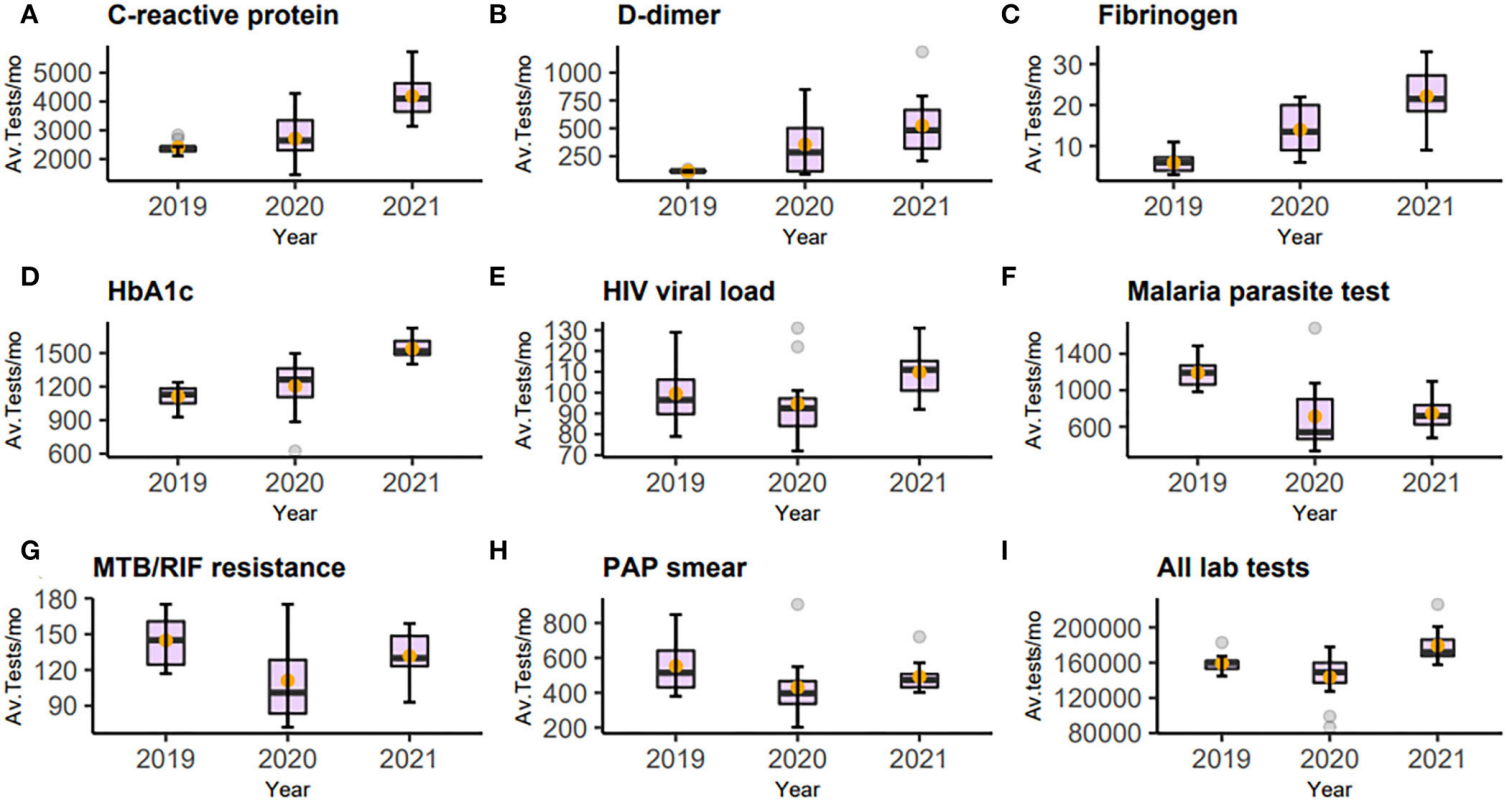
Figure of box-and-whisker plot **(A–I)** representing C-reactive protein, D-dimer, fibrinogen, HBA1c, HIV viral load, Malaria parasite test, MTB/RIF, PAP smear, and all laboratory tests. Each panel shows the changes in average tests per month (Av. Tests/mo) from 2019 to 2021 The mean is represented by the orange dot. The statistical summaries of these and other tests not plotted are shown in Table 1.

Analysis of laboratory tests revealed two major groups. The first was those whose test volumes decreased in the first year (2020) and then rose in the following year. Some recovered only marginally, but others to a level near the baseline, or in a few cases, surpassing the baseline. Included in this group malaria parasite test, MTB/RIF, blood group, blood count, and histology showed a statistically significant decrease. Blood count, a test included in diagnostic investigations, decreased by 15%, an average of 1,814 tests (95%CI: 397.2–3229.6; *p*-value 0.016). This was followed by only 6% growth for this test in 2021 compared to 2019 which was not statistically significant. On the contrary, the monthly average tests for malaria in 2020 decreased by 480 tests (95%CI: 218.9–740.4; *p*-value 0.001), translating to a 40.2% decline from 2019. This decrease persisted and was still statistically significantly lower than in 2019 (*p*-value <0.001). Other tests which showed a decrease, although not statistically significant, were serum sodium, PAP smear, blood culture, HIV viral load, and all laboratory tests summed together. The majority of these test orders showed variable recovery in 2021; however, test numbers for malaria and blood groups showed negligible recovery. The immediate explanation for this group was the restrictions implemented to control the spread of COVID-19 resulting in hospital avoidance. The second group was those whose test volumes increased despite restrictions both in the first and second year of the pandemic. These were thought to be caused by an increased utilization related to the clinical management of COVID-19. Included in this group were D-dimer, fibrinogen, CRP, and HbA1c. Tests for D-dimer, for instance, increased by more than four times from 2019 to 2021, an average of 114 to 525 (*p*-value <0.001). Orders for HbA1c initially showed a modest increment in 2020, but this rose by an average of 428 tests (95%CI: 346.6–508.2; *p*-value <0.001), translating to a 38.4% increment. Figure 2 and Table 1 show the graphical visualization and statistical analysis of the changes in the monthly test volume means in 2020 and 2021 compared to the baseline (2019).

**Table 1:** Table showing analysis of the changes in laboratory test in 2020 and 2021 compared to 2019 data.

	**2019**	**2020**			**2021**		
**Test**	**Monthly mean (Range)**	**Mean (Range)**	**Δ Mean [CI 95%]**	**t-statistic (*P-*value)**	**Mean (Range)**	**Δ Mean [CI 95%]**	**t-statistic (*P-*value)**
D-dimer	114 (83–135)	355.8 (89–849)	+240.8 [65.3–416.3]	3.02 (0.01)	525 (207–1187)	+411 [229.6–592.4]	4.98 (<0.001)
Fibrinogen	5.9 (3–11)	14 (6–22)	+8.1 [4.4–11.8]	4.64 (<0.001)	22.2 (9–33)	+16.3 [11.4–21.1]	7.16 (<0.001)
Malaria	1192.3 (983–1489)	712.7 (334–1,683)	−479.6 [218.9–740.4]	3.93 (0.001)	747.3 (478–1,098)	−445 [301.1–589.1]	6.41 (<0.001)
C-reactive protein	2401.8 (2,113–2,829)	2,712 (1,453–4,281)	+310.2 [0–225.2]	1.26 (0.23)	4186.9 (3,136–5,727)	+1785.1 [1268.3–2,302]	7.5 (<0.001)
HbA1C	1111.6 (928–1,239)	1204.1 (624–1,497)	+92.5 [0–72.2]	1.20 (0.25)	1,539 (1,401–1,723)	+427.4 [346.6–508.2]	10.97 (<0.001)
Cervical smear	553.6 (380–848)	430.6 (202–907)	−123 [0–262.5]	1.83 (0.08)	492.3 (402–721)	−61.3 [0–165.4]	1.24 (0.23)
MTB/RIF	144.8 (117–175)	111.2 (72–175)	−33.6 [6.6–60.6]	2.62 (0.02)	131.7 (93–159)	−13.1 [0–31.2]	1.49 (0.14)
HIV viral load	99.6 (79–129)	94.6 (72–131)	−5 [0–19.3]	0.72 (0.48)	109.8 (92–131)	+10.2 [0–2.2]	1.71 0.10
Serum Sodium	5242.3 (4,849–5,813)	4712.8 (2,339–5,631)	−529.5 [0–1,060]	2.15 (0.05)	5607.9 (5,104–6,587)	+365.6 [38.7–692.4]	2.36 (0.030)
Procalcitonin	736.4 (84–1,071)	733.8 (397–1,025)	−2.6 [0–185.2]	0.03 (0.98)	916.9 (616–1,289)	+180.5 [0–16.4]	1.90 0.07
Blood group	388 (333–441)	324.5 (229–520)	−63.5 [15.4–111.6]	2.83 (0.013)	353.8 (315–387)	−34.2 [11.9–56.4]	3.19 (0.004)
Blood count	11717.8 (10,660–14,100)	9904.3 (6,247–13,098)	−1813.5 [397.2–3229.6]	2.74 (0.016)	12479.5 (10,448–16,766)	+761.7 [0–445.6]	1.34 (0.19)
Blood culture	457.8 (428–518)	415.5 (279–578)	−42.3 [0–95.5]	1.71 (0.11)	484.1 (426–621)	+26.3 [0–13.2]	1.41 (0.17)
Histology	1407.9 (1,173–1,668)	1254.3 (785–1,450)	−153.6 [10–296.8]	2.23 (0.037)	1374.7 (1,179–1,658)	−33.2 [0–157.5]	0.55 (0.58)
All lab tests	159,385 (144,776–182,872)	143416.3 (86,696–177,800)	−15968.8 [0–34,165.3]	1.89 (0.08)	179385.2 (157,600–226,159)	+20000.2 [6945.2–33055.1]	3.24 (0.005)

The monthly mean test numbers and range for each year are shown. The differences in mean (Δ Mean) have been calculated, and increase or decrease compared to 2019 is depicted using (+) or (–) symbols, respectively. To test for statistical significance in the change, unpaired t-test, assuming unequal variance was applied. The t-statistic, corresponding 95% confidence interval (CI 95%), and p-values are presented. The last row (All lab tests) represents the monthly mean number of all tests performed in the laboratory including those not analyzed individually.

**Figure 3:**
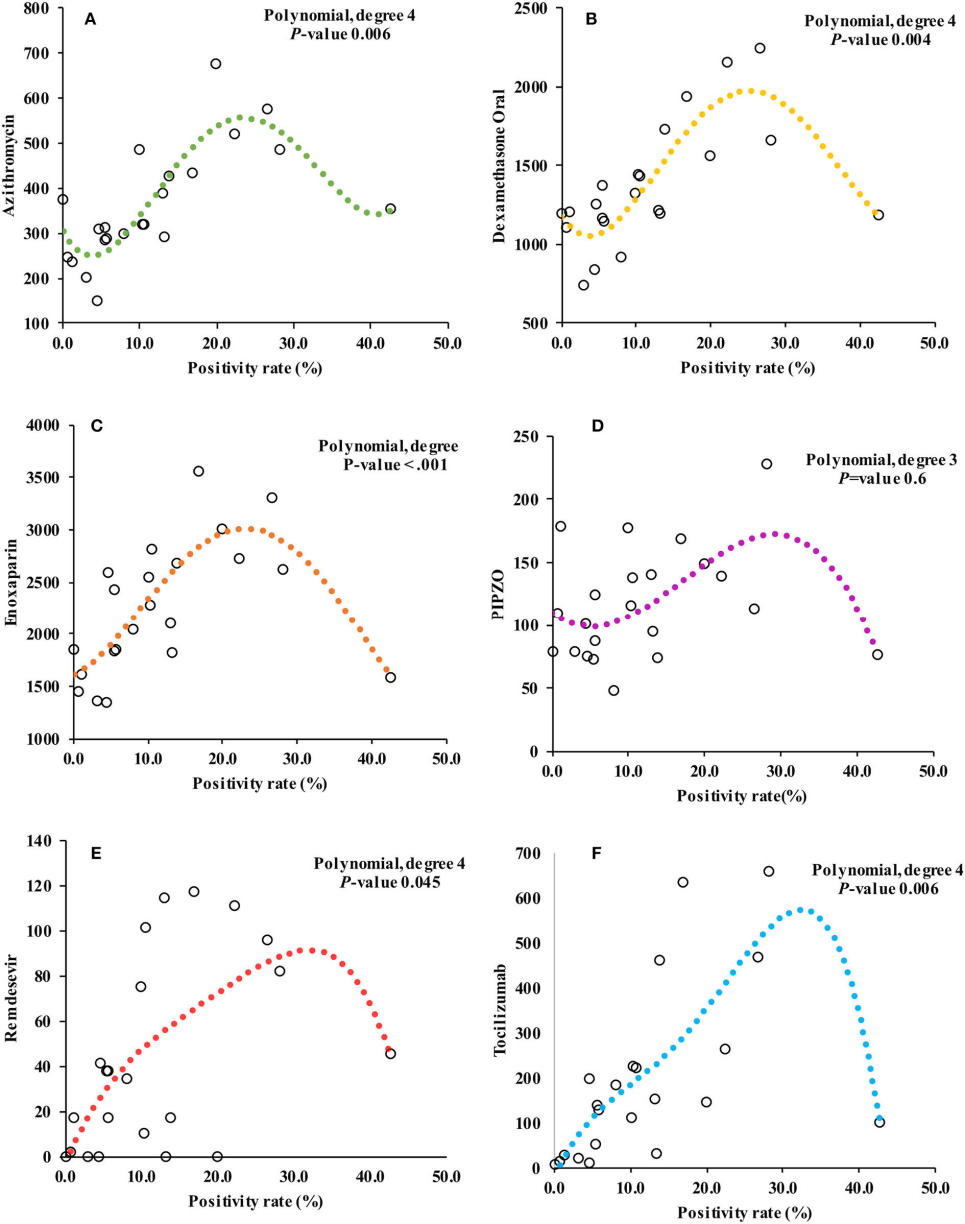
Figure showing the relationship between positivity rate and monthly prescription drug utilization. **(A–F)** represent the models for azithromycin, oral dexamethasone, enoxaparin, piperacillin/tazobactam (PIPZO), remdesivir, and tocilizumab, respectively. The models are non-linear, and polynomial regression best fitted a quadratic model with the exception of PIPZO, **(D)** which best fitted a cubic model. The *P*-values for each model shown are the outcome of partial F-test to determine whether there was a statistically significant difference from the linear model. The model for PIPZO was not significant. Not shown, are models for fentanyl (quadratic model, *p*-value 0.03) and intravenous dexamethasone (cubic model, *p*-value 0.04).

Pharmacy data also revealed a gradual to sharply increased utilization of the selected prescription drugs. As visualized in Figure 1, the utilization of dexamethasone, tocilizumab, and remdesivir peaked during the third (*Alpha* variant) and fourth (*Delta* variant) waves that saw positivity rates of 28.1 and 16.8%, respectively. Although there was an increase in utilization during the *Omicron* variant wave, which had the highest positivity rate (42.6%), it was still less compared to the previous two waves. The models for prescription drug utilization during the 2 years followed a non-linear regression model in relation to the positivity rate as shown in Figure 3. Oral dexamethasone, tocilizumab, enoxaparin, azithromycin, and remdesivir best fit a quadratic model (polynomial regression, degree four). The models were better than the linear models, with a partial F-test showing significant *p*-values ranging from 0.045 in the case of remdesivir to <0.0.001 in the case of enoxaparin, and the R^2^ values ranged from 0.31 in the case of PIPZO to 0.72 in the case of azithromycin. The regression for PIPZO best fit a cubic model; however, this was not any better than the linear model (*p*-value 0.6). Fentanyl (model not shown) best fit a quadratic model but was only marginally significant (*p*-value 0.036). The model for intravenous dexamethasone, unlike oral dexamethasone, was only marginally significant (*p*-value 0.047) on a cubic model (model not shown). In this study, we did not distinguish usual-care thromboprophylaxis from therapeutic-dose anticoagulation in regard to enoxaparin. The antibiotic PIPZO, typically used in the ICU setup, remdesivir, fentanyl, an anesthetic adjunct, showed weak predictive models. Tocilizumab, whose utilization was low in pre-COVID-19 times, increased with rising cases. A common feature noted for the pharmacologic agents was that the utilization was highest between March and August 2021, when *Alpha* and *Delta* were the predominant circulating variants. By the end of December 2021 which was the cutoff for our data collection, the *Omicron* wave was still ongoing and was the highest in terms of the number of cases and positivity rate. This was, however, characterized by a markedly reduced utilization, compared to the previous waves. The impact of the *Omicron* variant wave may therefore not be fully evident.

## Discussion

Laboratories regularly use data on test volumes and results to determine among other laboratory operations, reagent management, and workflow. The same is true for other departments in a healthcare facility whose aims are to meet the demands of testing, diagnosis, treatment, or prevention. These data, if carefully analyzed, may, however, be utilized for optimizing healthcare operations at a public health level. In this study, we have demonstrated that there was significant upward demand for CRP, fibrinogen, D-dimer, and HBA1c test in relation to rising cases and care of COVID-19 patients. Other laboratory tests were variably affected potentially due to social–economic factors. The demand for COVID-19-related therapeutics was best predicted by quadratic regression models. The highest demand for both tests and therapeutics was recorded during the *Alpha* and *Delta* variant waves of the pandemic.

One finding that has also been observed by other investigators was that interventions influenced by social political and public health concerns determined health-seeking behavior during the pandemic. Various degrees of restrictions and lockdowns likely lead to loss of income, hospital avoidance, fear of contracting COVID-19, postponement of elective surgical cases, and restricted travel (8, 21). In our study, the pattern for the malaria parasite test was perhaps an example where restricted movement changed testing demand and, by extrapolation, disease epidemiology. Nairobi, which is the county the study was conducted in, is not endemic to malaria (22). Typically, people contract malaria when they travel to malaria endemic counties, and therefore, a restricted movement was likely to lead to the further reduced incidence in Nairobi and hence reduced testing requirements. This observation may not be true for malaria endemic regions and for other diseases endemic in distinct geographic distributions.

There is no doubt that social restrictions were beneficial in managing COVID-19 patient numbers, especially until other interventions, such as vaccines, were widely available. However, one can see the potential negative effect of loss of continuity in care due to prolonged restrictions, for example, diseases such as tuberculosis (TB) and HIV whose prevalence in Africa is still high. Initial diagnosis and follow-up rely heavily on MTB/RIF and HIV viral load testing, and any lapse in the diagnosis, treatment, or follow-up would lead to loss of the benefits accumulated by the TB and HIV control programs (23). A study conducted in Nairobi, Kenya, implemented real-time monthly surveillance of TB and HIV activities to counteract the feared negative impact on TB and HIV programs. Small successes in treatment, follow-up, and referral were registered, showing the usefulness of more active intervention (24). This trend and potential intervention may also apply to cervical cancer screening which is dependent on PAP smears and human papillomavirus (HPV) testing. In the majority of African countries, screening uptake is already suboptimal, and this is in addition to East Africa having the highest cancer of the cervix disease burden (25). Loss of screening momentum is therefore a drawback in reducing the incidence of invasive cancer of the cervix. A late-stage cancer diagnosis is also a recurring health problem but is likely more common in Africa (26, 27). Therefore, decreased histology services could potentially mean delayed surgeries and diagnosis including cancer diagnosis and a worsening of late the stage cancer diagnosis problem.

Another pattern of interest was HbA1c testing. Testing for HbA1c is typically performed as a diagnostic test for diabetes or to monitor glycemic control over the last 3 months. Increment in HbA1c testing appears to be intuitive, given the fact that diabetes is a leading risk factor for severe COVID-19. However, the rapid rise points to COVID-19 unmasking a significant population with undiagnosed diabetes in the community. In a recent publication from our institution, including 913 records of admitted patients, the proportion of COVID-19 patients with diabetes at baseline was 27.3% and this rose by over 20% over the course of admission, bringing the proportion with diabetes to 48.1% (28). Given that glucocorticoids are essential for hospitalized patients with COVID-19, pre-diabetics who were tipped over to overt diabetes may have contributed to this proportion.

The interaction between pathology, pharmacy, and clinical care needs clearly emerged in our analysis. Hemostasis appears to have been a major issue given the simultaneous rise in prescriptions for enoxaparin and increased demand for fibrinogen and D-dimer test especially when hospitalization was high. Inflammation and immune dysregulation also were critical areas given the concurrent rise in inflammatory marker testing (CRP and procalcitonin) and inflammation modifying agents (dexamethasone and tocilizumab). Notable was the relatively modest increase in the utilization of prescription drugs during the *Omicron* variant wave which was the highest in terms of positivity rate. This provided some evidence that the *Omicron* variant was associated with less severe disease than the previous variants, with contributing factors being vaccination and immunity acquired *via* natural infection (29, 30). This observation should, however, be interpreted with the caveat that the impact of the *Omicron* variant may not have fully emerged since this wave was still ongoing at the cut-off time for our data collection. At an operations level, this pattern of decreasing utilization means that the demand for drugs used for COVID-19 patients and tests pushed up by COVID-19-related testing would decrease. Rationally, one would commensurately decrease procurement to avoid the other extreme problem of overstocking and expiry leading to healthcare resource wastage.

The study was limited by the lack of patient-level integration and analysis of the results of tests included in this study. The trends of test positive MTB/RIF, malaria, or out-of-range HbA1c, for instance, would provide more granular data on the translation of diagnostics to clinical care and treatment. In addition, data on which patients had what test, for what diseases, and what treatment was given would provide key information on the extent to which healthcare needs are met by a particular program, assess adherence to good clinical practice, and facilitate national healthcare planning. Furthermore, this was a targeted analysis; we only looked into a small subset of pathology, pharmacy, or nursing outputs. A comprehensive and unbiased analysis would, of course, require more sophisticated computational capacity. Although this was a targeted, single-institution study, we assume that we have demonstrated the power of integrated data analysis would have, when applied at a national level for the purposes of healthcare planning in Africa.

Data analytics has become a major tool in the study of global social and economic matters. Laboratories produce vast amounts of data; unfortunately, most of these data lie unanalyzed and therefore unusable by the community. These data are not only important for the estimation of disease prevalence but can also be indicators of access to treatment, follow-up, and unmet clinical needs. For example, during the first two waves, stock outs of laboratory reagents and consumables disrupted operations and by extension clinical care. This was occasioned by the abrupt changes in demand for tests outlined above combined with the need to process large numbers of COVID-19 tests. We, therefore, utilized the data we had collected, and the national epidemiological projections to inform the projected third wave. This prompted a targeted stocking of essential drugs, laboratory, reagents, and consumables resulting in better preparedness, a significant reduction in stock outs, and reduced disruptions in testing and healthcare delivery in the subsequent waves.

The results of this study inform us of the expected scenario in the event of other outbreaks with similar pathophysiology as COVID-19. Furthermore, they lead us to recommend increased efforts at diabetes screening, cervical cancer screening, and cancer screening as well as active follow-up of HIV and TB patients who may have discontinued follow-up. Harnessing data analytics is probably as important as investing in technology and human resource to improve pathology in Africa. With regard to future prospects, the wealth of information from this limited analysis makes a strong case for expanding its scope. The data that we have collected and analyzed will be an excellent resource for pathology and other clinical departments since other health data analytical projects can plug into this base, and the process can be amplified by multicenter partnerships. This will also be a stepping stone to “big data” analysis which is promising enormous potential even in healthcare (31). Investing in data information systems that can be seamlessly interlinked with other clinical public health departments, real-time analytics, and contributing to policy formulation will be a double-edged sword that would enable pathology departments to exert a broader impact in public healthcare.

## Data availability statement

The original contributions presented in the study are included in the article/Supplementary material, further inquiries can be directed to the corresponding author/s.

## Author contributions

AN, JK, JG, and DN: conceptualization. AN: retrieval pathology data, data analysis, visualization, and manuscript writing. JK and DN: retrieval and clean-up and analysis of hospitalization data. JG: retrieval and clean up and analysis of pharmacy data. All authors contributed to the article and approved the submitted version.

## Conflict of interest

The authors declare that the research was conducted in the absence of any commercial or financial relationships that could be construed as a potential conflict of interest.

## Publisher's note

All claims expressed in this article are solely those of the authors and do not necessarily represent those of their affiliated organizations, or those of the publisher, the editors and the reviewers. Any product that may be evaluated in this article, or claim that may be made by its manufacturer, is not guaranteed or endorsed by the publisher.

## Footnotes

^1^ https://coronavirus.jhu.edu/map.html

^2^ https://www.health.go.ke/
